# Inhalation for Consumption

**Published:** 1856-08

**Authors:** 


### INHALATION FOR CONSUMPTION.

IN our July number we denied the claim of *Inhalation* to the increased health of the country; especially did we oppose the idea that the diminished mortality from consumption was owing to the curative effects of medicated inhalation, because in other cities, where inhalation had not been introduced, the mortality had decreased.

The diminished mortality, from all diseases, of 1855, in comparison with 1854, although the population had increased, was for

New York, eighteen per cent.

Philadelphia, twelve per cent.

Boston, nine per cent.

Baltimore, three per cent.

By this showing, it appears that New York mortality was one third less than that of Philadelphia, being as eighteen to twelve. Does that difference in favor of New York arise from the fact that during 1855 medicated inhalation flourished like a green bay tree, while for the same year it was unknown in Philadelphia? It is rather difficult to prove a negative. It is known to intelligent men, that fewer foreigners came to New York in 1855 than came in 1854 by scores of thousands, and that it is from the death of these emigrants New York appears by statistical tables to be more unhealthy than it really is. Very many of these foreigners reach this city in a diseased and dying condition.

From this same cause—the diminution of foreign emigration—the diminution of the number of deaths from consumption is properly attributed. For, in round numbers, of the three thousand persons who died of consumption in New York city during 1854, nineteen hundred, nearly two thirds, were foreigners; and when we remember that perhaps two thirds of the inhabitants of New York are natives of the United States, the relative proportion of foreigners who die of consumption above the natives, is very great—at least seventy per cent.

To expose the fallacious claims of Inhalation further, it is sufficient to state, that the increase of mortality for 1854 above 1855, was owing to the fact that thousands died of cholera in 1854, which, as we all know, renders consumptive diseases more speedily fatal.

All that can be properly said in reference to the diminished mortality of New York in 1855, and the practice of medicated Inhalation, is, that it was merely a coincidence. Such changes occur in the history of all large cities; for example, in round numbers,

THE INTERMENTS IN PHILADELPHIA, during the first half of eight successive years, is as follows:

| | | | |
|---|---|---|---|
| 1849 | . . . 3800 | 1853 | . . . 4700 |
| 1850 | . . . 3800 | 1854 | . . . 5100 |
| 1851 | . . . 4000 | 1855 | . . . 5000 |
| 1852 | . . . 5200 | 1856 | . . . 5400 |

From this table it will be seen that the first half of 1853 gave 500 less deaths than 1852, and that the first six months of the present year gives a mortality of 400 over the same time in last year, although medicated inhalation has been introduced into Philadelphia since February last, by agents from head quarters in New York, to say nothing of the multitude of outsiders, who practice it independently. Now it would be as great a violation of honorable truth to say that the increased mortality of Philadelphia for the first half of 1856 was in consequence of medicated inhalation, as to say that the diminished mortality of New York for 1855, as compared with 1854, was the result of medicated inhalation. An honorable controversialist scorns the use of a doubtful argument, and would no more employ a mere coincidence for proof, than a brave man would strike a fallen foe.

There is no class of men in the world who would hail with more delight, any available remedy for consumption, or for any other disease, than educated physicians; it is as much against their nature not to do so, as it would be for a printer to oppose the employment of one of Hoe's presses. It is as much to the interest of the physician to lay hold of every new remedy for disease, provided it be an efficient one, as it is for an intelligent mechanic or farmer to take advantage of any of the labor-saving appliances which the industry of ingenious men is daily bringing to light; and with the same unmeasured contempt do they regard all men who endeavor to palm upon them, as an improvement, what is in reality not so—to palm upon them as a novelty what has been known for a century.

If there be any curative power in medicated inhalation, it will become known, and if it can cure consumption it will survive all opposition, as it ought to. But if it is available, why has one of the two principal inhalers of New York, who breathed alcohol, swallowed alcohol, and soaked in alcohol, for the cure of consumption, left the city permanently? If it is a cure, there is enough practice within ten miles of the city hall to make a dozen men as rich as they could desire. Or if a feeling of benevolence causes them to travel about for practice, we consider it an error of judgment, for New York is more easily accessible, and by a larger number of people, than any other spot in the country.

If medicated inhalation does cure consumption, where is the man of notoriety and education who has been cured by it, or who is willing to recommend it under his own signature?

If medicated inhalation is an efficient remedy for diseases ot the lungs, how is it that there is not a medical name, of any eminence, who has adopted the practice, *in any school?*

If, then, the intelligent men of the country cannot see practically or theoretically the advantages of this treatment, over others, the only rational conclusion to be arrived at is simply this—that it possesses no advantages above other forms of treatment, worthy of any special claim.

### CONSUMPTIVE EDITORS.

As a specimen of the means adopted to advance the claims of medicated inhalation, we append part of an editorial; whether the real author was the Inhalationist himself, and whether it was inserted for a consideration, and whether it was not intended to be copied into other papers for pay, are side questions.

"The fact is now too well established for dispute, that inhalation of medicated vapor, is the most natural, powerful and successful mode for consumption as yet reached—causing the absorption of tubercles, the cicatrization of cavities, and arresting the progress of disorganization of the lungs, when no other agency affords the least hope.

"We found a numerous representation of printers from all quarters of the country, owing to their peculiar liability to consumption.

"Of those who presented some remarkable cures, were different parties connected with the press of New York, as leading editors and proprietors. Through this we presumed, had they become identified with the advocacy of inhalation, in a manner which indicated an interest which nothing else could probably induce. Messrs. CLAYTON, of the Commercial Advertiser, JONES, proprietor of the Daily Times, BENNETT, of the Herald, and other similar persons in the press here, having been benefited by this new practice, gave it the impulse which it has had through that channel."

From seeing the above article, the general impression made on the mind of an ordinary reader is, that the persons there named were cured of consumption by means of medicated inhalation. But on a second reading, no such thing is pretended to. All that is really claimed is, that these persons were "BENEFITED" by the new practice.

That such a prominent paper as the New York Express should spare a quarter of a column in praise of the virtues of medicated inhalation, because it had merely '*benefited*' the persons there named, is most extraordinary. If every medicine or every doctor having claims to have "benefited" a patient, were praised at this rate, newspapers would have nothing else to do. But the point of the article, to which we wish to direct the reader's attention is, its criminality, if producing a false impression is criminal. It was adroitly written, and no doubt with much study, for the whole sentence, without saying so, leaves the impression on the mind of a casual reader, that among the "*remarkable cures*" effected by medicated inhalation, were the men whose names are given. But when the article is analyzed, the names of those who were "*benefited*" are very particularly mentioned, while the names of those who presented cases of "*remarkable cures*," and which really it was most important to know, are not given at all!

It is a shame, that men will lend themselves to such petty deceptions for a few dollars; and yet, so it is, and so it ever will be, until intellect and morals; until intelligence and truth; until cultivation and honesty, shall become one and inseparable.

